# Collecting Information on Caregivers’ Financial Well-Being: A Document Review of Federal Surveys in Canada

**DOI:** 10.1177/07334648221099279

**Published:** 2022-05-21

**Authors:** Husayn Marani, Sara Allin

**Affiliations:** 1 Institute of Health Policy, Management and Evaluation, Dalla Lana School of Public Health, 206712 University of Toronto, Toronto, ON, Canada; 2 North American Observatory on Health Systems and Policies, University of Toronto, Toronto, ON, Canada

**Keywords:** caregiving, policy, decision-making, social security, financial risk, document review, Canada

## Abstract

Population-based surveys conducted by governments inform strategies concerning emergent areas of policy interest. One such area is unpaid caregiving in the context of an aging population. In the Canadian and global contexts, research suggests a need for public financial support to mitigate financial risks of caregiving. In this document analysis, we reviewed 17 federal surveys since 2005 to understand how caregiving-related information is captured. We found that caregiving-related questions were largely derived from two surveys, the General Social Survey and the Canadian Community Health Survey. However, gaps exist concerning questions related to estimates of private care expenditure, and the impacts of older adult caregiving across domains of financial risk (income, productivity, and healthcare utilization). Addressing these gaps, either through revising existing surveys or a new national survey on unpaid caregiving, may improve meaningful assessments about risks and impacts of caregiving, which may better inform public strategies that offset these risks.

**What this paper adds**

• Population data collected by public (government) surveys informs public policy on emerging areas of policy interest
• As the global population ages, there is growing policy interest in ways to support unpaid caregivers; yet, in Canada specifically, the challenges of unpaid caregiving, particularly financial challenges, are poorly captured on existing public surveys

**Applications of study findings**

• Governments (in Canada and internationally) should consider including questions concerning financial challenges associated with unpaid caregiving on national surveys
• A fulsome understanding of financial risks of caregiving (e.g., out-of-pocket care–related expenditure and impacts on income and employment status) may better inform public policies to offset these risks
• Methods undertaken to review public documents (government surveys) are innovative and may inform similar studies concerning gaps in public data collection in different contexts (e.g., government, non-governmental organizations, and academia)

## Introduction

In 2020, expenditure on research and development represented 1.69% of gross domestic spending in Canada (OECD, 2022). Although Canada falls behind other OECD members, including the United States (U.S.) (3.07%) and the United Kingdom (U.K.) (1.76%), the federal government of Canada maintains a strong commitment to research, particularly public (government) data collection. In 2020, over half a billion dollars was spent on public data collection, an expenditure that is projected to increase by 16% next year (Statistics Canada, 2020a).

In Canada, collecting and disseminating public data is a federal responsibility, legislated by the *Statistics Act* (1985). Public data is collected nationally through a comprehensive network of surveys conducted by Statistics Canada, a federal agency. Although data from non-governmental organizations and academic research may inform public policy, public data collected directly by governments has an important role in shaping public policy decision-making, including raising awareness of issues, identifying target groups for specific policy interventions, informing the development of new policies, and justifying ongoing policy intervention across different public sectors (Hastak et al., 2001; Laws et al., 2013). In the Canadian context, information collected by Statistics Canada informs federal, provincial and territorial policy and programming, monitoring and surveillance, agenda setting, and financial benchmarks concerning employment and income, and is also used by researchers, analysts, and planners in both public and private settings (Statistics Canada, 2009).

Surveys conducted by Statistics Canada are conducted on a cyclical basis (e.g., annually or every 5 years) to capture data on emerging topics of national research and policy interest. In recent years, unpaid caregiving has become one such area. Unpaid caregiving refers to provision of medical, emotional, and/or psychological support over any duration of time for someone (e.g., family member, friend, or neighbor) living with a health condition or limitations in activities of daily living (“ADLs”), including bathing, toileting, dressing, eating, transferring (e.g., from chair to bed), and mobility (Arriagada, 2020). Across Canada, there were 7.8 million unpaid caregivers in 2018, representing 25% of the population over 15 (Arriagada, 2020). Alongside similar patterns observed in the UK, Belgium, and Austria, this represents one of the highest rates across OECD countries (OECD, 2021). The number of unpaid caregivers in Canada and worldwide is expected to rise, in part, due to an aging population and a growing desire to age independently at home rather than facility–based long-term care (Huber, 2021; March of Dimes Canada, 2021). These sentiments have been further stimulated by the COVID-19 pandemic (National Institute on Ageing, 2020a), the first wave of which saw 80% of deaths due to COVID-19 occurring in long-term care facilities (Webster, 2021).

Several analyses of unpaid caregiving using datasets from government surveys consisting of short supplements on unpaid caregiving (namely, the Canadian Community Health Survey [CCHS] and the General Social Survey [GSS]) have been conducted in the past decade. Findings expose a range of risks associated with unpaid care provision, particularly financial risks. We interpret financial risk to include both the magnitude of private expenditure on care (care expenses paid out-of-pocket), and the impacts of these expenses across various domains of financial risk, including productivity, income earning potential, and healthcare utilization (Hacker, 2004). Based on these analyses, financial risks may be rooted in deficits in publicly subsidized home care provision particularly in certain geographies such as rural areas (Kitchen et al., 2011). Other risks include caregivers exiting the formal, or paid, labor market, or modifying work hours and taking leaves of absence from work to provide unpaid care (Jacobs et al., 2013; Lilly et al., 2010; Stanfors et al., 2019), which is commonly experienced by caregivers in the context of “intensive caregiving”, such as end-of-life care (Williams et al., 2014). Caregivers may also experience a reduction in overall leisure and self-rated health (Stanfors et al., 2019).

Despite these analyses, important gaps remain concerning the financial risks of caregiving. For example, increasingly, local and international scholarship is demonstrating that unpaid caregivers are incurring costly care-related expenses out-of their own pocket, including expenses for prescription medication, supplies, housing, and transportation (Han et al., 2012; Shooshtari et al. 2017; You & Kobayashi, 2011). However, little is known about how these expenses manifest as financial risk based on analyses of government datasets. We know from empirical scholarship that cost-prohibitive expenses may be forcing key trade-offs between other important expenses such as food (Law et al., 2018). There is also a concern that stressors associated with paying for care-related expenses may contribute mental and physical health issues thereby increasing caregivers’ utilization of cost-prohibitive health services (Chambers et al., 2016). We also know how caregiving may interfere with gainful employment, thereby compromising income-generating potential and the ability to pay for important care-related expenses (Longacre et al., 2016).

The literature described above points to the importance of providing supports to caregivers; however, gaps in the literature call into question the conclusions we can draw from government surveys about the financial risks of unpaid caregiving. In the context of this paper, we refer specifically to public (government) support. Although some public caregiving benefits exist, including income-tested tax exemptions under the Disability Tax Credit (Government of Canada, 2021a), and Family Caregivers Benefits for employed individuals who take time off work (between 15 and 26 weeks) to provide care (Government of Canada, 2021b), these may not be wholly inclusive of all caregivers, especially those who are not employed and those providing care beyond the maximum eligibility period. Similar deficits in financial support (e.g., cash allowances and tax credits) in contexts such as the UK and US have been observed (Pattyn et al., 2021). Accordingly, there is an increasing need to improve the supports available to unpaid caregivers to offset the financial risks of caregiving and the impact of these risks on caregivers beyond the departure from formal (paid) labor force to provide care (National Institute on Ageing, 2020b). Indeed, supporting unpaid caregivers has important health system implications, as it keeps care recipients out of costly acute care and residential long-term care facilities, which is more cost-effective.

Hence, information obtained through government-administered surveys could help to inform caregiver groups who may need help mitigating financial risks, and therefore, better improve the design of policies to support unpaid caregivers and mitigate the financial risks of caregiving. Thus, in this paper, we explore the extent to which federally administered surveys capture information on unpaid caregiving across Canada. Specifically, we seek to understand whether questions and response options on existing survey instruments consider the full range of financial risks of unpaid caregiving (including care-related expenses) and their impact among unpaid caregivers.

## Methods

To address our research aim, we conducted a document review of population-based, federal survey instruments—and the survey questions therein—conducted by the Canadian federal government since 2005. We did not analyze completed survey datasets. Document analyses give voice and meaning around an assessment topic (Bowen, 2009). Documents reviewed may include public records (e.g., policy manuals or strategic plans), personal documents (e.g., e-mails, social media posts, first-person accounts), and physical evidence within study settings (e.g., flyers or handbooks) (O’Leary, 2014). In this document analysis, we apply approaches to reviewing documents to publicly administered surveys. To do this, we draw on O’Leary’s (2014) considerations for reviewing documents and conducting textual analysis where possible.

We focussed specifically on federal surveys as population-based data collection is a federal responsibility and provinces and territories routinely use federally collected data to inform local decision-making. While some Canadian provinces and territories also routinely collect data from their residents in surveys, the focus of our study was on federal government surveys given their capacity to shape policy across the country (Hastak et al., 2001; Laws et al., 2013) and to narrow the scope of this paper, but we recognize other potential sources of national data exist, including from non-governmental organizations and academic research.

### Eligibility and Inclusion

O’Leary (2014) suggests developing a list of texts to explore and considering how to access these texts. For the purpose of this analysis, this meant deciding which survey instruments we should extract questions from, and from where to locate these survey instruments. All survey instruments are housed on the “Surveys and statistical programs” page on the Statistics Canada website (statscan.gc.ca) under “Results and documentation of surveys and statistical programs.” In this analysis, questions from the most recently circulated survey within the past 15 years were extracted. Recently inactive surveys from the same time period were still included in this review, if salient, in the event that the types of questions asked differ from any active surveys.

Statistics Canada surveys cover 31 subject areas. Subject areas are not mutually exclusive as surveys may fall under multiple subject areas. Based on our overarching interest in unpaid care across the domains of financial risk, surveys from the following subject areas were reviewed for eligibility: “Families and Households” (*n* = 6 surveys); “Health” (*n* = 64); “Income, pensions, spending, and wealth” (*n* = 27); “Labor” (*n* = 74); “Seniors and aging” (*n* = 5); and “Society and community” (*n* = 15).

Akin to processes in collecting literature using databases for traditional literature reviews (Arksey & O’Malley, 2005), we determined eligibility of surveys to be extracted by independently conducting then comparing results from a manual search within each survey instrument for questions containing standard care- or caregiver-related keywords, including “care,” “caregiver,” “care provider,” “family,” “family member”, “family worker,” “assistance,” “unpaid,” “voluntary,” or “informal”. These keywords have been used in seminal literature reviews in this topic area (Queluz et al., 2020) and reflect best practices in inclusive language in caregiving-related research (Stall et al., 2019). If any of these keywords appeared in any question, the survey instrument was included in this review.

### Extraction of Survey Questions and Analysis

The extraction of survey questions from included survey instruments was completed by HM and validated by SA and consisted of two elements. First, we extracted details concerning the survey instrument itself, for example, its purpose and target audience. These details were derived from the detailed description of the survey instrument on the Statistics Canada website, which we adapted for brevity. Then, we extracted specific survey questions by reviewing all questions in each included survey instrument and coding relevant questions deductively based on an a priori conceptual understanding of the financial risks of caregiving. Questions were coded across three broad categories: (1) the [financial] risks of caregiving, including sub-categories such as (a) estimates of direct private (out-of-pocket) care expenditure, (b) sources of financial support, and (c) general spending behaviors); (2) consequences of unpaid care provision across specific domains of financial risk, including (a) income, (b) employment and productivity, and (c) health and health care; and (3) the determinants, or predictors, of these risks as described in relevant literature, including (a) dwelling, or the living arrangement of caregiver and care recipient, (b) care provision, or the type and extent of care provided and to whom, (c) employment status of survey respondent, and (d) personal income and income sources (Dosman & Keating, 2005; Guerriere et al., 2008; Leong et al., 2007). We excluded baseline demographic questions consistently asked across all household surveys (e.g., gender, ethnicity, age, and relationship status with care recipient), but recognize these, too, may inform patterns of financial risk.

## Results

This document analysis was conducted in December 2020. At this time, Statistics Canada had a collection of 412 active and 384 inactive (no longer in circulation) surveys. With the exception of the long-form census, all active and inactive subject-specific surveys from 2005 onward were considered for this review to capture the most recent versions of all possible survey instruments currently in circulation or now inactive. Following screening for eligibility and the removal of outdated survey versions, 17 survey instruments were included in this analysis, summarized and referenced in Table 1, and further described in Supplemental Appendix A.

**Table 1.** *Summary of Included Survey Instruments*.

Survey Name	Status (Active or Inactive)	Last Circulated	Subject Areas	Reference
Survey of household spending (SHS)	Active (Annual)	Jan 2, 2019–Feb 14, 2020	Expenditures; dwelling characteristics; household equipment; income	Statistics Canada, 2021b
General social survey (GSS)—caregiving and care receiving	Active (every 5 Years)	Apr 3–Dec 28, 2018 (cycle 32)	Care and social support; disability; health and well-being; society and community	Statistics Canada, 2018a
GSS—family	Active (every 5 Years)	Feb 1, 2017–Nov 30, 2017	Aboriginal peoples; education, training and learning; ethnic diversity and immigration; families, households and housing; health	Statistics Canada, 2019
GSS—volunteering and participating (GVP)	Active (every 5 Years)	Sept 4, 2018–Dec 28, 2018 (cycle 33)	Labor; society and community; unpaid work; volunteering and donating	Statistics Canada, 2015
Canadian community health survey (CCHS)	Active (every 2 years)	Jan 2–Dec 24, 2020	Disease and health conditions; health; health care services; lifestyle and social conditions; mental health and well-being	Statistics Canada, 2016
CCHS—Healthy aging supplement	Inactive (occasional	Dec 1, 2008–Nov 30, 2009	Health; health and disability among seniors; lifestyle and social conditions; population aging; population and demography; seniors	Statistics Canada, 2008
Canadian health survey on seniors (CHSS)	Active (occasional)	Jan 2–Dec 24, 2020	Diseases and health condition; health; health care services; lifestyle and social conditions; mental health and well-being	Statistics Canada, 2020d
Participation and activity limitation survey—adults, 15 and over (PALS)	Inactive (replaced by Canadian survey on disability)	Oct 30, 2006–Feb 28, 2007	Disability; equity and inclusion; health; society and community	Statistics Canada, 2007
Canadian survey on disability (CSD)	Active (every 5 years)	Mar 1, 2017–Aug 31, 2017	Disability; equity and inclusion; health; society and community; work, income and spending	Statistics Canada, 2018b
National household survey (NHS)	Inactive (one-time)	May 10, 2011–Aug 24, 2011	Aboriginal peoples; education, training and learning; ethnic diversity and immigration; families, households and housing; income, pensions, spending and wealth; labor; languages; population and demography; society and community	Statistics Canada, 2011a
Survey on living with neurological conditions in Canada (SLNCC)	Inactive (one-time)	Sept 9, 2011–Mar 21, 2012	Diseases and health conditions; health	Statistics Canada, 2011b
Employment insurance coverage survey (EICS)	Active (annual, 4 5-week collection cycles)	Apr 17, 2018–Feb 15, 2019	Employment insurance, social assistance and other transfers; labor; non-wage benefits	Statistics Canada, 2020c
Survey of older workers	Inactive (one-time)	Oct 19, 2008–Dec 1, 2008	Labor; work, transitions and life stages	Statistics Canada, 2010
Labour force survey (LFS)	Active (monthly)	Apr 2020	Employment and underemployment; hours of work and work arrangements; industries; labor; occupations; unionization and industrial relations; wages, salaries and other earnings	Statistics Canada, 2021a
Canadian survey of economic well-being (CSEW)	Inactive (one-time)	Aug 18, 2013–Oct 7, 2013	Household, family and personal income; income, pensions, spending and wealth; low income and inequality	Statistics Canada, 2013a
Survey of labour and income dynamics (SLID)	Inactive (formerly annual. Since merged with Canadian income survey)	Jan 1, 2011–mid-Mar (over 6-year period)	Families, households and housing; household, family and personal income; income, pensions, spending and wealth; labor; low income and inequality	Statistics Canada, 2013b
Survey of financial security (SFS)	Active (occasional)	Sept 8, 2016–Dec 8, 2016	Household assets, debts and wealth; income, pensions, spending and wealth	Statistics Canada, 2020b

### Description of Survey Instruments

All included surveys were cross-sectional in nature (no panel surveys were included in our search). Six survey instruments are inactive because they were circulated only one-time, or amalgamated with, or replaced by, another instrument. One survey instrument, the GSS, is conducted annually but reflects a different theme every year, which is conducted on a 5-year cyclical basis. For this reason, three versions of the GSS are included in this review, representing three thematic areas—“Caregiving and Care Receiving,” “Family,” and “Volunteering and Participating.”

Statistics Canada defines a dwelling as distinct physical living quarters with a private entrance outside, and a household is any person or group of persons living within a dwelling (Statistics Canada, 2020e). For the most part, all surveys are targeted toward all households. Some surveys specifically target respondents of other household surveys. For example, participants of the Labour Force Survey (LFS) are sub-sampled to participate in a variety of related surveys, including the Survey of Older Workers and the Survey of Financial Security. Although the GSS on Caregiving and Care Receiving the Healthy Aging Supplement of CCHS include short modules targeting unpaid caregivers, no survey solely targets unpaid caregivers. Thus, survey respondents may either be care recipients, or caregivers responding on their own behalf or as a proxy for a care recipient living within the same household.

Surveys span a number of subject areas including income, social support, education, labor, and workforce participation, health, and family and living arrangement. Participation in all surveys is voluntary with the exception of the LFS which is mandatory under the Statistics Act (Statistics Canada, 2021a). Based on the data extracted concerning stated objectives of surveys, survey data may be used for a variety of purposes, including adjusting payments by or benefits from various social programs, calculating financial benchmarks (e.g., Consumer Price Index and Gross Domestic Product), calculating spending behaviors at the individual and household level, identifying needs across specific groups (e.g., older adults and low-income communities), for general monitoring, surveillance and evaluation, and to assist in future decision-making concerning policy initiatives (e.g., concerning employment rate).

Based on the description of these surveys, it appears that one of the 17 surveys—the GSS on Caregiving and Care Receiving—is used to develop policy and programming for unpaid caregivers, but this description does not explicitly identify how survey results from previous cycles have informed policy and program development.

### Description of Survey Questions

We then analyzed the questions within these 17 survey instruments to understand how survey instruments explored the financial risks of unpaid caregiving. Supplemental Appendix B synthesizes relevant questions across all survey instruments, which we summarized and adapted for brevity. Questions may directly or indirectly concern the provision of unpaid care for someone with a health conditions or limitations in ADLs. Questions may also target unpaid caregivers or otherwise. Questions were organized (coded) across the categories described in our Methods. In some cases, the relevance to unpaid caregiving is not obvious in the question itself, but in response options. In such cases, relevant response options are italicized in Supplemental Appendix B. As we were broadly interested in the types of questions asked across all survey instruments, and there is duplication in questions across federal surveys, we did not aim to identify from what survey instruments each question was derived.

Table 2 presents a tally of survey instruments that consist of at least one question corresponding to a category described in our Methods. Across all categories, there were 13 instances across two survey instruments where at least one question is asked of an unpaid caregiver respondent about the provision of unpaid care for someone living with a long-term health condition or limitations in ADLs (■). Most such questions were asked in the GSS survey on Caregiving and Care Receiving, introduced for the first time in 2018, and another, now inactive, one-time Healthy Aging supplement of CCHS. Among these questions, there is interest in the type and duration of care provided by unpaid caregivers, the broad cost implications (e.g., total out-of-pocket costs of care across all care provided), and employment implications.

**Table 2.** *Tally of Questions Captured Across all Included Survey Instruments*.

Survey Name	Predictors				[Financial] Risks of Caregiving			Consequences of Caregiving		
	Dwelling	Care provision	Employment/activity status	Personal income and income sources	General spending behaviors (care-related)	Financial support available for caregiving	Estimates of monetary costs (direct, private) of caregiving/care receiving	Income-related consequences	Employment/productivity consequences	Health and quality of life consequences
Survey of household spending (SHS)	●		●	●	●		●			
General social survey (GSS)—caregiving and care receiving	■	■	■			■	■		■	■
GSS—family	●		●		○	●	○		○	●
GSS—volunteering and participating (GVP)		●	●	●		●			●	●
Canadian community health survey (CCHS)	●	□	●	●		●			□	●
CCHS—Healthy aging supplement	■	■	□	□		■	■		■	■
Canadian health survey on seniors (CHSS)	●	□								●
Participation and activity limitation survey—adults, 15 and over (PALS)	□	□	□			●	●	●		●
Canadian survey on disability (CSD)		□					●	●		
National household survey				●			○			
Survey on living with neurological conditions in Canada (SLNCC)	●	□	□	●			●	●	□	
Employment insurance coverage survey (EICS)		○	●	●		●	○	○	○	
Survey of older workers			□	●				●	□	
Labour force survey (LFS)			□							
Canadian survey of economic well-being (CSEW)				●				●		
Survey of labour and income dynamics (SLID)	●		●	●	●	●	○		●	●
Survey of financial security (SFS)			●	●		○		●		

*Notes*: (■) denotes at least one question that concerns the provision of unpaid care for someone living with a long-term health condition or limitations in activities of daily living that is asked specifically of a respondent who may be an unpaid caregiver (i.e., survey has a caregiving module). (□) denotes at least one question (and/or response option) that concerns the provision of unpaid care for someone living with a long-term health condition or limitations in activities of daily living, but the question does not explicitly target an unpaid caregiver (e.g., targeted at a care recipient where question is framed in the context of care received). (●) denotes at least one general question (unrelated to caregiving and not asked of an unpaid caregiver or care recipient), but may be relevant to caregiving in that question could apply to, or be adaptable to an unpaid caregiving context. (○) denotes at least one question that concerns the provision of childcare only (survey respondent is a parent/guardian).

We also included questions that may be relevant to caregiving (as a predictor of financial risk) but do not expressly target unpaid caregivers. Across all categories, there were 15 instances across eight survey instruments of at least one question (or response option) concerning the provision of unpaid care (□). In these cases, the question was framed in the context of care received and were largely targeted toward respondents who are recipients of care or assistance for a health condition. For example, several surveys (CCHS, CHSS, CSD, and SLNCC) ask care recipients about the type of care they receive (personal care, transportation, scheduling, etc.), which may be provided by an unpaid caregiver. This information helps to understand the prevalence of unpaid care, the characteristics of the care being provided, and the carer.

Also included on this tally are general questions that are agnostic to caregiving, but, based on an a priori understanding of the caregiving experience, may be relevant in analyses of these datasets (●). Across all categories, there were 50 such instances across 14 survey instruments. Questions largely concerned predictors of the caregiving experience; for example, employment status, personal income, and spending behaviors. These questions are not specific to the caregiving experience, but have been noted here because they may be adapted to fit a caregiving context. For example, questions concerning outstanding spending behaviors (bills and debts, current mortgages on assets, general reliance on credit cards to meet regular expenses, etc.) are noteworthy if caregiving contributes to such debts, or if such debts compromise funds available to pay for care.

Lastly, we included 10 questions across five survey instruments concerning the provision of childcare where the survey respondent is a parent or guardian (Ο). Such questions could be adapted for unpaid caregiving of someone living with a health condition or limitations in ADLs.

## Discussion

The purpose of this analysis is to broadly understand what is being asked about the provision of unpaid care on government-administered survey instruments in Canada with a focus on national surveys administered by Statistics Canada. Our review found 17 survey instruments that related, in some way, to unpaid caregiving. Of these, 10 asked questions specifically about the experiences and risks of caregiving irrespective of target audience. And of these, only two instruments expressly targeted unpaid caregivers—the GSS on Caregiving and Care Receiving and the CCHS Healthy Aging Supplement, neither of which are currently active or in circulation. The majority of survey instruments, while not explicitly targeting respondents who are unpaid caregivers, present questions that are useful in understanding predictors of financial risk if any respondents are unpaid caregivers, and could be adapted to fit the caregiving experience.

Across surveys, there are notable gaps in questions concerning financial risks of unpaid caregiving. For example, while PALS is specifically concerned with non–reimbursed out-of-pocket care expenses, it is no longer active and its successor, CSD, does not ask questions concerning out-of-pocket expenditure. While surveys such as the GSS on Caregiving and Care Receiving ask unpaid caregivers to estimate total out-of-pocket care-related expenditure, little is known about what constitutes total expenses, limiting targeted public approaches to minimize specific expenses that may be most cost-prohibitive. This calls into question whether current policy and programming on unpaid care—for example, tax exemptions under the Disability Tax Credit (Government of Canada, 2021a), and generosity of coverage in financial risk protection programs such as the Family Caregivers Benefit (Government of Canada, 2021b)—are informed by the most up-to-date data. Relatedly, although some surveys ask respondents whether or not they use public financial support, questions concerning the specific type of support and extent and magnitude of support are missing, limiting our understanding of whether support programs like the Family Caregivers Benefit are effective at offsetting financial hardships of caregiving.

Furthermore, private expenditure data, largely derived from SHS, is used to inform Canada’s National Health Expenditure Framework, but, as it stands, it is impossible to know the extent to which caregivers themselves incur private health expenditure in the provision of unpaid care as SHS does not explicitly target unpaid caregivers. Again, this limits our understanding of the financial impacts associated with unpaid caregiving, and may limit policy decisions concerning financial risk protection, including compensation schemes and grants.

In terms of income, and employment and productivity, LFS asks one question about the main activity of respondents who report not being in the formal labor force. In this question, all responses are informal (unpaid) activities, including childcare, older adult care, household work, and unpaid internship. Generally speaking, however, surveys related to labor and employment (see Table 1) do not include questions on informal work, and questions concerning earnings and earning potential are about formal labor participation; other than time spent across all activities, the monetary value of informal, or unpaid, labor is not measured. Echoed in relevant scholarship from the U.K. (Aldridge & Hughes, 2016), this raises a broader question of how Canada conceptualizes the informal labor workforce, and the extent to which informal labor should be captured in labor-specific instruments of data collection. This is important because unpaid caregivers represent an increasingly growing segment of the informal workforce worldwide (Broese van Groenou & De Boer, 2016), and unpaid caregivers of those living with complex health conditions like dementia—the rate of which is growing in Canada—are seeing themselves departing the formal workforce to accept unpaid care responsibilities without the workplace and government benefits afforded to parent caregivers (Black et al., 2010; Longacre et al., 2016). On this note, surveys appear to present a bias toward leaving formal employment to care for infants and young children. Few surveys are concerned specifically with productivity and income implications of caring for those living with health conditions or limitations in ADLs, a highly heterogenous population characterized by very diverse caregiving activities and support needs (Young et al., 2020).

Lastly, while we know income is a determinant of health, this connection is not reflected in any health- or quality of life-related questions targeting unpaid caregivers specifically—for example, the extent to which care-related losses in income (departing the formal labor force) may have compromised the ability to pay for personal health expenditure, which has been observed elsewhere (Broese van Groenou & De Boer, 2016).

While literature is scant in both the Canadian and international contexts concerning how government surveys ask about caregiving, previous scholarship has observed that persons receiving long-term care and their caregivers are not well-represented in national surveys administered by Statistics Canada (Hirdes et al., 2018). Outside Canada, research from the U.K. suggests that, while the U.K. Census and Family Resources Survey do include questions on caregiving, the monetary value of caring is poorly captured because caregiving is a personal activity that generally takes place in the home, whereas government surveys are concerned with measuring economic activity in specific markets and public sectors, such as the labor market (Aldridge & Hughes, 2016). Similar findings are echoed in the U.S., where there is no uniform approach in national surveys to ascertain the number of unpaid caregivers, their attributes and services provided, thereby compromising public policy regarding unpaid caregiving for older adults (Giovannetti & Wolff, 2010).

### Implications

Accordingly, this study has important implications on research, policy and practice both in Canada and internationally. Beyond the way results from government surveys have been shown to shape best practices in protecting caregiver employers (AARP, 2016), we know that government surveys may inform the policy-making process, including agenda setting (Hastak et al., 2001; Laws et al., 2013). For example, findings from social surveys administered by the federal government inform debates in public policy about important policy topics such as pension reform, which can translate directly into new or revised policies (McDonald, 1997). In the context of aging and caregiving, demographic trends in chronic disease prevalence captured by CCHS revealed over 700,000 Canadians living with Alzheimer’s Disease or related dementia, a statistic that spurred the passing of Bill C-233—*An Act respecting a national strategy for Alzheimer’s disease and other dementias*, in 2017.

With the federal government budgeting $41.3 million over 6 years for Statistics Canada to improve data infrastructure and data collection on health care, chiefly supportive care, primary care and pharmaceuticals (Department of Finance Canada, 2021)), there may be value in orienting future research and/or policy action on developing a specific survey on unpaid caregiving, or a regularly occurring, enhanced version of GSS on Caregiving and Care Receiving, that fills gaps identified in this study. In particular, we recommend addressing gaps concerning out-of-pocket care expenditure, including assistive devices and formal (paid) home care support, how caregiving has impacted income-generating potential, the extent to which public supports may have offset risks to gainful employment, and financial risk has manifested during COVID-19, which exacerbated the stressors of caregiving, including maintaining full-time employment (Seedat & Rondon, 2021).

From a methodological perspective, the adaptation of document review methods to analyze federal government survey instruments is innovative and could inform similar studies in other settings across different orders of government. Indeed, unpaid caregiving is a globally ubiquitous topic area and insights from this study could give rise to similar studies in other contexts. A variety of trends across Canada and abroad suggest a greater need for governments to publicly collect information that elucidates the experiences of unpaid caregivers and the relevant impacts, particularly the financial impacts. One important trend is the shift from institutional to community-based or home care through “Aging-at-Home” or “Aging-in-Place” strategies, occurring in jurisdictions across several countries with national health insurance systems such as Canada, Australia, and the U.K. (Australian Government Department of Health, 2021; Government of Ontario, 2010; Sixsmith & Sixsmith, 2008). These strategies were developed to shift the economic burden of the aging population away from publicly funded acute and long-term care systems. Inadvertently, however, this shift may be imposing greater care and financial responsibilities on unpaid caregivers, which is inconsistent with perspectives on social welfare distribution in welfare states that otherwise accept the responsibility to provide (publicly pay for) health and social care. Hence, from a policy and practice perspective, collecting precise information on the implications of this shift on caregivers is important in determining whether the state should intervene in better protecting caregivers from any financial risks of caregiving.

### Limitations

We focussed on government data collection on the financial risks of caregiving, but note that academia, industry and non-governmental organizations have vested interests in this topic area. Future lines of inquiry could review how data is collected in these sectors and how it is used to develop policies and best practices that offset the financial risks of caregiving. We also excluded baseline demographic questions common across all surveys, but recognize that demographic questions such as relationship between caregiver and care recipient, living arrangement, age, and household income are important predictors of financial risk.

## Conclusion

We found that the federal government of Canada is asking many of the right questions concerning unpaid caregiving. However, due to the increased role of unpaid caregivers, and to inform policies and programs, the Canadian government should consider more focused and frequent surveys that assess the financial risks and impacts of caregiving on unpaid caregivers and their households. In a post–COVID-19 world where we may see a rapidly increasing role of unpaid caregivers, results of this study may be useful not only in Canada but elsewhere where unpaid caregivers will represent a large segment of the informal workforce.

## Supplemental Material


Supplemental Material - Collecting Information on Caregivers’ Financial Well-Being: A Document Review of Federal Surveys in Canada
Supplemental Material for Collecting Information on Caregivers’ Financial Well-Being: A Document Review of Federal Surveys in Canada by Husayn Marani and Sara Allin in Journal of Applied Gerontology

Supplemental Material - Collecting Information on Caregivers’ Financial Well-Being: A Document Review of Federal Surveys in Canada
Supplemental Material for Collecting Information on Caregivers’ Financial Well-Being: A Document Review of Federal Surveys in Canada by Husayn Marani and Sara Allin in Journal of Applied Gerontology
