# Supplementary material for: Collecting Information on Caregivers’ Financial Well-Being: A Document Review of Federal Surveys in Canada
Source: J Appl Gerontol. 2022 May 21;41(9):2033–44. doi: 10.1177/07334648221099279 (PMC9434210; doi:10.1177/07334648221099279)
Supplement: Supplemental Material - Collecting Information on Caregivers’ Financial Well-Being: A Document Review of Federal Surveys in Canada [file sj-pdf-1-jag-10.1177_07334648221099279.pdf]

Supplementary Appendix A. *Summary of Federal Surveys Administered by Statistics Canada Included in Review*

Survey Name	Purpose	Status (Active or Inactive)	Last Circulated	Subject Areas	Participation/ Sample Frame	Data Collection	Response Rate	Tax Record Linkage	Survey Use Based on Description on Statistics Canada website
**Survey of Household Spending (SHS)**	Collect information on amounts spent on food, clothing, shelter, transportation, health care, other items; also dwelling characteristics and household furnishings and equipment.	Active (Annual)	Jan 2, 2019 – Feb 14, 2020	Expenditures; dwelling characteristics; household equipment; income	Voluntary; 21,250 households across Canada	Personal interview and diary of daily expenses	Provinces: interview 66.8% and diary 41.3%<br><br>Territories: interview 64.4% and diary 33.7%	Yes	1) Update calculation of Consumer Price Index (CPI), used to adjust payments on social programs (CPP, OAS, CCB), and wage and salary settlements;<br>2) assist in calculation of GDP;<br>3) measure how much households spend on basic expenses (shelter, food, clothing);<br>4) show trends in household spending on education and health care;<br>5) help government agencies identify needs of low-income, senior-citizen and lone-parent households;<br>6) compare spending habits of different types of households (low-income, senior citizen, lone-parent, single member households, etc.);<br>7) monitor housing adequacy, suitability and affordability
**General Social Survey (GSS) - Caregiving and Care Receiving**	Collect information on Canadians who provide care to family and friends living with long-term health conditions, and on Canadians who receive this care, including challenges both groups face that are of current or emerging interest.	Active (Every 5 Years)	April 3- Dec 28, 2018 (Cycle 32)	Care and social support; disability; health and well-being; society and community	Voluntary; sample frame N/A	Electronic questionnaire and telephone interview.	52.8%	Yes	1) Results used by analysts and researchers to study current situations and trends, and by government departments to develop/design policies and programs that can have an impact on individuals who receive care, their families who provide care, and those who may need or provide care in the future.
**GSS - Family**	Monitors changes in Canadian families by collecting information on conjugal and parental history (chronology of marriages, common-law unions and children), family origins, children's home	Active (Every 5 Years)	Feb 1, 2017 – Nov 30, 2017	Aboriginal peoples; Education, training and learning; Ethnic diversity and immigration;	Voluntary; 43,000 households	Computer-assisted telephone interviewing	20,000 (52.4%)	Yes	The information collected will impact program and policy areas such as parental benefits, child care strategies, child custody and spousal support programs.

	leaving, fertility intentions, and other socioeconomic characteristics.			Families, households and housing; Health					
**GSS - Volunteering and Participating (GVP)**	To collect national data concerning individual contributory behaviours including volunteering, charitable giving and civic participation; 2) to provide reliable and timely data to the System of National Accounts; 3) to inform both the public and voluntary sectors in policy and program decisions that relate to the charitable and volunteer sector.	Active (Every 5 Years)	Sept 4, 2018 – Dec 28, 2018 (Cycle 33)	Labour; Society and community; Unpaid work; Volunteering and donating	Voluntary; 40,000 households	Computer-assisted telephone interview.	24,000	Yes	N/A
**Canadian Community Health Survey (CCHS)**	Gather health-related data (health status, health care utilization, health determinants) at the sub-provincial levels of geography (health region or combined health regions)	Active (Every 2 years)	Jan 2 – Dec 24, 2020	Disease and health conditions; health; health care services; lifestyle and social conditions; mental health and well-being	Voluntary; 65,000 respondents across Canada	Computer-assisted telephone and personal interviews	N/A	Yes	1) Support health surveillance programs by providing health data at the national, provincial and intra-provincial levels.
**CCHS – Healthy Aging Supplement**	CCHS supplement that collects new information from Canadians 45+ about the factors, influences and processes (e.g. health, social and economic determinants) that contribute to healthy aging.	Inactive (occasional)	Dec 1, 2008 – Nov 30, 2009	Health; Health and disability among seniors; lifestyle and social conditions; population aging; population and demography; seniors	Voluntary; 32,005 respondents across Canada (sub-sampled from 2006 Census)	Computer-assisted telephone and personal interviews	30,865	**No**	To help policymakers, researchers planners make informed decisions regarding health care, social services and income support programs for Canadians. Specific objectives include: 1) To better understand the aging process of people aged 45 and over by collecting data on various aspects of their health and well-being, use of health care services, social support and participation and work and retirement transitions; 2) to examine how lifestyle determinants affect health as people age; 3) to examine how lifestyle determinants affect health as people age; 4) to examine how lifestyle determinants affect health as people age

**Canadian Health Survey on Seniors (CHSS)**	CCHS supplement that collects information on health status, health care services, supports, and social and health determinants for the Canadian population aged 65 and over.	Active (Occasional)	Jan 2 – Dec 24, 2020	Diseases and health condition; health; health care services; lifestyle and social conditions; mental health and well-being	Voluntary; ~25,000 (10,000 additional dwellings and 15,000 from CCHS sample)	Computer-assisted telephone interview and personal interview	N/A	No	1) To better understand what contributes to healthy aging; 2) to produce estimates on the health of seniors aged 65 and over at the provincial level, and for seniors aged 85 and over at the national level; 3) to produce a cross-sectional dataset on the health of seniors that permits analysis on a range of research questions and surveillance activities; 4) to evaluate the changes on certain aspects of health from CCHS; 5) to help policy makers, researchers and planners make informed decisions regarding health care, social services and support programs for the aging population and that will affect all Canadians.
**Participation and Activity Limitation Survey - Adults, 15 and Over (PALS)**	Gathered information about Canadian adults and children whose everyday activities may be limited because of a health-related condition or problem, and the barriers they face in all areas of life (home, work, school, communities).	Inactive (replaced by Canadian Survey on Disability)	Oct 30, 2006 – Feb 28, 2007	Disability; Equity and inclusion; Health; Society and Community	Voluntary; 47,793 people across Canada based on responses to 2006 Census of Population Questionnaire	Computer-assisted telephone interview (questionnaire)	8,954 children with proxies (<15), 38,839 adults (>15)	No	1) Data used by all levels of government for numerous programs, including the development of employment programs, paying for aids and assistive devices such as wheelchairs and Seeing Eye dogs, support services, new social program development and health, leisure and recreation programs; 2) to evaluate policies and programs that affect Canadians with activity limitations, and to help develop new policies and programs throughout Canada.
**Canadian Survey on Disability (CSD)**	To provide information about Canadian youth and adults whose everyday activities are limited due to a long-term condition or health-related program.	Active (every 5 years)	March 1, 2017 – Aug 31, 2017	Disability; Equity and Inclusion; Health; Society and Community; Work, income and Spending	Voluntary; 50,000 sampled from question on 2016 Census of Population long form	Internet-based Electronic Questionnaire (self-reported questionnaire completed online by respondent and interviewer-led method conducted by	40% (self-reporting), 60% telephone interview	No	1) To help all levels of government, as well as associations for persons with disabilities and researchers better plan and evaluate services, programs and policies for Canadians living with disabilities to help enable their full participation in society; 2) to support effective development and operation of the Employment Equity Program; 3) to fulfill Canada's international agreement relating to the United Nations Convention on the Rights of Persons

						telephone)			with Disabilities
**National Household Survey**	Designed to provide information about people in Canada by their demographic, social and economic characteristics, as well as provide information about the housing units in which they live. Information formerly collected by mandatory long-form census questionnaire.	Inactive (one-time)	May 10, 2011 – Aug 24, 2011	Aboriginal peoples; Education, training and learning; Ethnic diversity and immigration; Families, households and housing; Income, pensions, spending and wealth; Labour; Languages; Population and demography; Society and community	Voluntary; 4.5 million households	Questionnaire (2 modes: paper or (mail delivery) and online.	N/A	No	To provide data to support federal, provincial, territorial and local government planning and program delivery.
**Survey on Living with Neurological Conditions in Canada (SLNCC)**	To assess the impact of chronic neurological conditions (~18 including migraine, headaches, multiple sclerosis, epilepsy and Alzheimer's disease), on quality of life, work and general well-being for individuals with these conditions; to evaluate the costs associated with these conditions including out-of-pocket expenses and loss of productivity for individuals with these conditions; to analyse the impact of caring on families, and caregivers.	Inactive (one-time)	Sept 9, 2011 – March 21, 2012	Diseases and health conditions; health	Voluntary; 8,200 people (sampled from CCHS)	Computer-assisted telephone interview	81.6%	No	1) To provide a clear picture of neurological conditions in Canada; 2) to help the Public Health Agency of Canada, Health Canada, and provincial ministries of health better plan and provide health services and programs for people with chronic neurological conditions; 3) to help researchers monitor, analyse, measure and report on factors affecting those living with chronic neurological conditions.
**Employment Insurance Coverage Survey (EICS)**	To study coverage of the employment insurance program, and provide a meaningful picture of who	Active (Annual, 4 5-week collection	April 17, 2018 – Feb 15, 2019	Employment insurance, social assistance and other transfers;	Voluntary; sample frame N/A (sampled from LFS)	Telephone interviews based on computerized	N/A	N/A	1) to produce a series of precise measures to identify groups with low probability of receiving benefits; 2) to provide a detailed description of the

	does or does not have access to EI benefits among the jobless and those in a situation of underemployment; also covers maternity and parental benefits.	cycles)		labour; non-wage benefits		questionnaire conducted within seven weeks of lst LFS interview			characteristics of the last job held as well as reasons for not receiving benefits or for not claiming.
**Survey of Older Workers**	To assess the labour market intentions and transitions of older Canadians, including factors that influence decisions to retire or remain working and other intentions and motivations (pensions, finances, role od dependents, nature of work, health considerations)	Inactive (one-time)	Oct 19, 2008 – Dec 1, 2008	Labour; work, transitions and life stages	Voluntary; sample frame N/A (sub-sample of LFS)	Computer-assisted telephone interviewing	79.5%	N/A	1) To support labour policy initiatives in the deferral government; 2) to inform policy development; 3) to assist in future decision-making
**Labour Force Survey (LFS)**	One of four surveys that characterize labour market events in Canada. LFS provides employment estimates by industry, occupation, public and private sector, hours worked, etc. For employees, data on wage rates, union status, job permanency and establishment sized produced.	Active (Monthly)	April 2020	Employment and underemployment ; hours of work and work arrangements; industries; labour; occupations; unionization and industrial relations; wages, salaries and other earnings	Mandatory; sample frame 56,000 households	Series of 6 interviews across 6 months. Computer-assisted telephone interviewing based on computerized questionnaire or personal visit from field interviewer	100,000 individuals	N/A	1) to produce the well-known unemployment rate and other standard labour market indicators such as the employment rate and participation rate; 2) Used by different levels of government for evaluation and planning of employment programs in Canada; 3) Regional unemployment rates used by Employment and Social Development Canada to determine eligibility, level and duration of insurance benefits for persons living within a particular employment insurance region; 4) Data also used by labour market analysis, economists, consultants, planners, forecasters and academics in both the private and public sector.
**Canadian Survey of Economic Well-Being (CSEW)**	To produce estimates of the incidence of goods or activities that are lacking at the national and provincial geographies, at the household level to examine the basic economic needs and financial	Inactive (one-time)	Aug 18, 2013 – Oct 7, 2013	Household, family and personal income; Income, pensions, spending and wealth; Low income and	Voluntary; 24,000 households (sampled from LFS)	Computer-assisted telephone interviewing	67% (country average)	Yes	The information will be used by Employment and Social Development Canada, Statistics Canada and other research organizations interested in improving well-being to assess the needs of households in Canada.

	circumstances of households in Canada.			inequality					
**Survey of Labour and Income Dynamics (SLID)**	Complements traditional survey data on labour market activity and income with an additional dimension: the changes experienced by individuals over time. At the heart of the survey's objectives is the understanding of the economic well-being of Canadians: what economic shifts do individuals and families live through, and how does it vary with changes in their paid work, family make-up, receipt of government transfers or other factors?	Inactive (Formerly annual. Since merged with Canadian Income Survey)	Jan 1, 2011 – mid-March (over 6-year period)	Families, households and housing; Household, family and personal income; Income, pensions, spending and wealth; Labour; Low income and inequality	Voluntary; Two panels consisting of two LFS rotation groups (17,000 households each). Panel surveyed for a period of six consecutive years. New panel introduced every three years, so two panels always overlap.	Computer-assisted telephone interviewing	67.3%	Yes	The survey data are used by federal (Human Resources and Skills Development Canada, Finance, Canada Mortgage and Housing Corporation , etc.) and provincial departments to formulate social policies and programs. Non-government organizations, private consultant firms and academics also use SLID data to do research to support their positions and to lobby governments for social changes. Individuals and families can use the data to compare their earnings and income situations with those of similar types of family compositions.
**Survey of Financial Security (SFS)**	Provides a comprehensive picture of the net worth of Canadians. Information is collected on the value of all major financial and non-financial assets and on the money owing on mortgages, vehicles, credit cards, student loans and other debts.	Active (Occasional)	Sept 8, 2016 – Dec 8, 2016	Household assets, debts and wealth; Income, pensions, spending and wealth	Voluntary; N/A (derived from LFS area frame)	Computer-assisted telephone interviewing	21,112 dwellings, 13,328 dwellings selected from the urban strata and 7,784 dwellings within 696 clusters from the rural strata.	Yes	The survey data are used by government departments to help formulate policy, the private sector and by individuals and families to compare their wealth with those of similar types of families.
