# Supplementary material for: Collecting Information on Caregivers’ Financial Well-Being: A Document Review of Federal Surveys in Canada
Source: J Appl Gerontol. 2022 May 21;41(9):2033–44. doi: 10.1177/07334648221099279 (PMC9434210; doi:10.1177/07334648221099279)
Supplement: Supplemental Material - Collecting Information on Caregivers’ Financial Well-Being: A Document Review of Federal Surveys in Canada [file sj-pdf-2-jag-10.1177_07334648221099279.pdf]

Supplementary Appendix B. *Synthesis of Question Types*

Category	Summary of Questions Across Included Survey Instruments (Relevant Response Options *Italicized*)	
	Questions specifically targets unpaid caregivers	Questions do not target unpaid caregivers, but may predict caregiving risks if respondent is an unpaid caregiver
**Living arrangement and dwelling habitants ("Dwelling")**	• living arrangements of caregiver (*e.g. same household or community; live-in full-time or part-time, or remote; and whether or not caregiver moved closer or moved in with care recipient*)<br>• distance travelled by caregiver to care recipient's hope (including method of travel, time to travel, cost of travel)<br>• previous living arrangement of care recipient in institutional care facility (*e.g. hospital, long-term care, nursing home care, private residential care*)<br>• relationship, age and sex of additional members of dwelling (including additional care recipients and/or dependents)	• type of dwelling and number of bedrooms<br>• the need for renovations, remodelling or additions<br>• how dwelling is financed (*rented or owned with or without financing*)<br>• dwelling ownership<br>• physical accessibility (*e.g. steps, ramp, doorways, grab bars, elevator*) or technological accessibility (*e.g. monitoring devices, smart home technology, sensors, cameras*) to facilitate daily activities and improve capacity to care<br>• number of people living in the dwelling<br>• whether care for someone is a reason for moving into, back into, or close to care recipients' home
**Care provision**	• profile of care recipient (defined as someone living with a long-term health condition (lasting longer than or expected to last longer than six months), including: physical or mental disability, problems related to aging and its severity (*mild, moderate or severe*);<br>• the type of care provided (*e.g. transportation to medical appointments, meal preparation, house cleaning, laundry, outdoor work, personal care (dressing changes, eating, bathing, toileting), medical care (preparing medications, medical treatments, dressing changes), scheduling or coordinating care-related tasks, managing finances, using medical equipment or supplies*	• health conditions of care recipient (as respondent): arthritis, osteoporosis, cardiovascular disease, kidney disease, asthma, chronic bronchitis, diabetes, migraine, back problems, cancer, mental illness, dementia, other neurological disease, urinary or bowel incontinence, digestive disease, fibromyalgia, chronic fatigue, developmental disabilities, injuries from an accident<br>• preferences by care recipients of unpaid family care versus professional care<br>• receipt of short-term or long-term assistance at home, work or school from family, friends or neighbours because of a health condition<br>• type of assistance received (*e.g. personal care such as assistance with eating, dressing, bating or toileting,*

	(wheelchair, pads for incontinence, ventilator))<br>• length of time care has been provided<br>• number of care recipients needing assistance<br>• relationship of caregiver to care recipient(s)<br>• average time spent providing care within specific time periods (*e.g. hours, days, weeks*)<br>• overcoming challenges in unpaid caregiver's capacity to provide care (*e.g. receipt of help from paid workers, family members or friends, and frequency of this help)*<br>• difficulties in finding additional help or qualified assistance and why *(e.g. did not know where to look, expensive, etc.)*)	*medical care, managing care such as making appointments or managing personal finances, childcare, transportation, meal preparation, emotional support, etc.*)<br>• if individual who provides assistance works at a job or business<br>• if individual who provides assistance works at a job or business at the same time as providing assistance<br>• if individual who provides assistance reduced hours or stopped work to provide assistance which lasted more than three months
**Employment/ activity status**	• general employment status (*e.g working, not working, retired, etc.*)	• current occupation of primary caregiver (*e.g. self-employed or otherwise*)<br>• work status (*e.g. employed, unemployed, retired, full-time, part-time*)<br>• length of time worked within given periods, including weeks in a year, days in a week, hours in a day
**Personal income and income sources**		• income before taxes and deductions from employment/self-employment (*e.g. personal income from wages, salaries, bonuses, tips, commissions, and farm/non-farm activities*), government, employer/private pension; investment; and other sources<br>• income from government programs and employment Insurance benefits (*e.g. maternity and parental benefits; Veterans' Pensions; welfare payments; benefits from Canada or Quebec Pension Plan; Disability Benefits included in Canada or Quebec Pension Plan benefits (CPP/QPP); Old Age Security Pension and Guaranteed Income Supplement; Provincial or municipal social assistance or welfare*

		(agnostic to caregiving); Child Tax Benefit, universal child care benefits, or family allowances (federal, provincial and territorial), etc.)<br>• income from employer or private pension sources (*e.g. regular pension income from an employers' pension plan payments from RRSP annuities or RRIF annuities; other job-related retirement pensions, including superannuation and annuities; and workers' compensation*)<br>• Investment sources (*e.g. dividends and interest on bonds or savings, accounts, Guaranteed Income Supplement (GICs) and mutual funds, and rental income (excluding capital gains or losses)*)<br>• income from other sources (*e.g. child support payments, spousal support (alimony), scholarships; and inheritance*)
**General spending behaviours (caregiving)**		• if respondent is financially supporting the care of a family member<br>• the length of time the financial support provided for the care of a family member is expected to last<br>• outstanding bills and debts, including current mortgages on assets, as well as general reliance on credit cards to meet regular expenses<br>• extent to which personal, daily and other various expenses (*e.g. mortgage, groceries, child-related care and clothing, electricity, heating, telephone, cable, internet bills*) are shared among household members<br>• extent to which money is available after paying for essential expenses<br>• extent to which savings are used and in what contexts<br>• expenses that are cut, reduced or delayed to pay for other expenses
**Financial**	• if there is another person who could provide	• types of financial support or assistance received for

**support available for caregiving**	assistance in times of challenge	(provision of care or otherwise)<br>• from whom financial assistance was received (*e.g. an individual; private or employer-sponsored insurance plan; government program such as tax reduction or welfare; family member*)<br>• additional supported needed (financial or otherwise)<br>• types of financial support provided to others<br>• general ability to pay bills on time<br>• reasons if affordability is a reason bills cannot be paid on time<br>• reliance, if any, on other individuals for financial needs (*e.g. for housing, food, etc*.)
**Estimates of monetary costs of caring (direct, private) of caregiving/care receiving**	• total direct costs (out-of-pocket and costs reimbursed) for care received<br>• regular payments made on behalf of a family member living with care recipient<br>• if cost is a barrier for purchasing caregiver supports<br>• how care services were obtained (*e.g. government program, private agency, family member/friend/neighbour, volunteer organization, etc*.)<br>• how expenses are paid for<br>• value for money (satisfaction) in paid home care provision<br>• reason for expenses incurred (*e.g. purchasing items such as medications or medical supplies; paying for services such as housekeeping or daycare; increases in expenses such as additional housing costs or food; transportation such as gas or parking*)	• direct costs related to: dwelling (*e.g. property; school taxes; property insurance; condominium fees; mortgage payments; electricity/hydro/sewage; rent; repairs and renovations; technological upgrades; hired labour for home upgrades*); technology (*e.g. smart technology, television, internet, cell phones; wearable technology*); childcare outside home; food/clothing; transportation (*e.g. accessible vehicle and gas or public transit expenses*); health care if not reimbursed (*e.g. drugs, assistive devices, dental exams, procedures and dental products, vision exams, procedures and products, allied care*); general practitioners and other specialists (e.g. *uninsured services such as renewals, diagnostic tests, services in private clinics*); premiums (*e.g. for private health insurance, dental insurance, disability insurance); financial services (planning, tax preparation, accounting*); hairdressing and grooming services; housekeepers (*including cleaners and house-sitters*); outdoor groundskeeping (*e.g. lawn maintenance, snow and garbage removal*); and home supports

		• personal out-of-pocket expenses (*e.g. prescription and non-prescription medications, assistive devices like mobility aids or specialized equipment, rehabilitation therapies, home care services*)
**Income-related consequences**	• consequences if income is not enough to cover care expenses (*e.g. having to skip or delay a non-mortgage debt payment, borrow money from a family member, friend or pay-day loan, declaring bankruptcy or making a formal proposal or informal financial arrangements to creditors, picking up another job to supplement costs*)	• consequences on retirement status (*e.g. coming out of retirement and pulling from retirement income/savings*)<br>• financial difficulty because of a long-term disability or health problem of household member<br>• mitigating financial hardship (*e.g. asking for financial help for friends or family for day-to-day expenses; taking on debt or selling an asset; turning to a charity organization (such as a food bank or thrift store*)
**Employment/ productivity-related consequences**	• changes in main activity among caregivers (e.g. paid work) at the start of caregiving experience (*e.g. leaving paid employment full- or part-time to provide care*)<br>• why time off work was not taken (*e.g. could not afford to, afraid to lose job, employer barriers, alternative care arrangements by friends and family, found acceptable [day] care services, preferred to work, workplace flexibility, etc.*)<br>• decisions not to return to work at all (*e.g. caring for a specific period of time, paid to take leave, obligations to stay home, cannot find or afford care, cannot find suitable job, etc.*)<br>• care arrangements upon returning to work (managed both, shared care with someone else, paid private home supports, employer-based daycare, public home supports at a reduced cost)<br>• incentives to return to paid workforce (*e.g. improved health, caregiving no longer required, job will always be available, etc.*)	• main activity if not working (*e.g. caring for children, household work, going to school, travelling, volunteering, unpaid internship, caregiving other than for children, illness or disability, retired*)<br>• reasons for workplace absences (*e.g. caregiving for young child or older adult, preference to stay at home, semi-retirement, began school, work was not suitable, long-term illness, desire to volunteer, working another paid job, injury etc.*)<br>• reasons for job departure, work absences, reduction in work hours, work unavailability (*e.g. own illness or disability, caring for own children, caring for an elder relative, etc.*)<br>• reasons for retirement (*e.g. improved health, providing care to a family member, etc.)*<br>• length of time of workplace absences<br>• reasons for return to work (*e.g. caregiving duties not required anymore*)<br>• receipt of any income from employer or otherwise during these absences

	• employment support for caregiving or otherwise (*e.g. paid leave, allowing flexible work schedule, providing transportation to get to work, etc.*)	• subsequent return to work after these absences<br>• extended reduction in paid work hours or shift to part-time work
**Health and quality of life-related consequences**	• strain on relationships as a result of caregiving<br>• health behaviours (*e.g. levels of exercise and changes in eating habits as a result of caregiving*)<br>• physical strain on body from caregiving<br>• visits to health professionals for personal health problems emerging from caregiving<br>• injuries as a result of caregiving<br>• emotional stress: *stress levels and sources of stress such as managing needs of, declining health of, and conflicts with, care recipient*<br>• coping strategies (*e.g. exercising, professional help, etc.*)<br>• access to respite support (planned relief or assistance) for caregiver	• self-rated health (*e.g. eating habits, physical activity levels, stress, sleep, feelings, emotion, disability screening, long-term health conditions*)<br>• how health concerns (*e.g. surgeries, injuries, ailments, etc.*) have compromised continued ability to perform daily activities (*e.g. caregiving*)<br>• unmet personal care needs<br>• barriers to accessing health and health care needs<br>• help-seeking behaviours (*e.g. reasons for not seeking information about treatments, services or therapies to improve own health*)<br>• participation in social, recreational, or group activities, and desires/barriers to participating in such activities<br>• foregoing medication because of costs
